# Body Concept and Quality of Life in Patients with Idiopathic Dystonia

**DOI:** 10.3390/brainsci10080488

**Published:** 2020-07-28

**Authors:** Lejla Paracka, Florian Wegner, Claus Escher, Martin Klietz, Martina de Zwaan, Mahmoud Abdallat, Assel Saryyeva, Joachim K. Krauss

**Affiliations:** 1 Department of Neurology, Hannover Medical School, 30625 Hannover, Germany; Wegner.Florian@mh-hannover.de (F.W.); escher.claus@mh-hannover.de (C.E.); Klietz.Martin@mh-hannover.de (M.K.); 2 Center for Systems Neuroscience, 30625 Hannover, Germany; Krauss.Joachim@mh-hannover.de; 3 Department of Psychosomatic Medicine and Psychotherapy, Hannover Medical School, 30625 Hannover, Germany; dezwaan.martina@mh-hannover.de; 4 Department of Neurosurgery, Hannover Medical School, 30625 Hannover, Germany; abdallat.mahmoud@mh-hannover.de (M.A.); saryyeva.assel@mh-hannover.de (A.S.)

**Keywords:** dystonia, body concept, quality of life, depression

## Abstract

Patients with dystonia experience unusual postures and disfigurement. The aim of the study was to examine changes in the body concept in relation to quality of life and severity of dystonia. Our cohort consisted of 20 patients with idiopathic dystonia resistant to medical therapy who were planned for pallidal deep brain stimulation. The results were compared to 25 healthy controls. The patients were assessed with Frankfurt Body Concept Scale, Short Form 36 (SF-36) Health Survey, Hamilton Depression Scale, Beck Depression Inventory, Social Phobia Inventory and Social Interaction Anxiety Scale. The disease severity was evaluated with Burke–Fahn–Marsden Dystonia Rating Scale and Toronto Western Spasmodic Torticollis Rating Scale. Patients with dystonia had a significantly impaired body concept in eight out of nine subscales in comparison to healthy controls. The differences were most pronounced for the subscales general health, body care, physical efficacy, sexuality and physical appearance (*p* < 0.001). Furthermore, all eight subscales of SF-36 exhibited significantly lower values in patients with dystonia compared to controls. We also found significant positive correlations between SF-36 and body concept subscales. Impairment of body concept was not associated with disease severity or levels of social anxiety symptoms. However, there was a significant association between self-rated depression and disease severity. Our patients suffered from increased depression and social anxiety symptoms except social interaction anxiety. We conclude that patients with dystonia have significant body concept impairment that interferes with quality of life in both physical and emotional domains. Future studies should focus on assessing these symptoms after adequate therapeutic management of motor symptoms.

## 1. Introduction

All dystonias are classified along two axes: clinical characteristics and etiology [1,2]. Clinical characteristics represent the phenomenology of dystonia in a patient. It includes age of onset, body distribution (e.g., generalized, segmental, focal), temporal pattern and association with other disorders. The second axis outlines the etiology of dystonia covering anatomical changes (degeneration, structural change or neither) and pathogenic disease patterns (inherited, acquired and idiopathic). Primary dystonias include inherited and idiopathic dystonias. Secondary dystonias manifest from other disease states or brain injury.

Involuntary movements and twisted postures in dystonia may cause substantial disfigurement. This may lead to disability in the patients’ everyday life and dissatisfaction with physical appearance. Embarrassment due to physical appearance may interfere with social interaction and cause social avoidance and isolation [3,4,5].

Patients with dystonia also have a higher risk for depression and anxiety, which may further affect quality of life [5,6,7,8]. While several studies have demonstrated impaired quality of life in several domains [9,10,11,12], the subjective perception of stigmata and disfigurement has received little attraction [13].

Feeling unattractive and apologetic specifically about their appearance has been shown in patients with cervical dystonia [14]. However, whether there are wider perceptions of body concept alterations has not been fully clarified yet. The aim of our study was to determine the dimensions of body concept alterations and associated changes in quality of life in patients with dystonia as compared to healthy controls. We also investigated possible associations of body concept alterations with measures of depression and anxiety and the severity of dystonia.

## 2. Materials and Methods

The study population comprised 20 adult patients with dystonia (10 men aged 51.2 ± 6.6, 10 women aged 55.5 ± 7.2, mean ± standard error of the mean (SEM)) with a mean disease duration of 11.5 ± 2.3 years [15]. Eight patients had generalized dystonia, five segmental dystonia and seven isolated cervical dystonia. Two patients were diagnosed with DYT-TOR1A (formerly DYT1) hereditary generalized dystonia, while dystonia in the other 18 patients was idiopathic. In all instances, botulinum toxin did not provide substantial long-term relief after long-term injections, and they were scheduled for pallidal deep brain stimulation [16]. Exclusion criteria for the present study were a diagnosis of acquired or combined dystonia and cognitive impairment. Mini Mental State Tests (MMSTs) [17] were performed in all patients to evaluate cognitive impairment. Severity of dystonia was determined with the Burke–Fahn–Marsden Dystonia Rating Scale (BFM) [18] and the Toronto Western Spasmodic Torticollis Rating Scale (TWSTRS) [19]. Patients were further asked to complete the Frankfurt Body Concept Scale (Frankfurter Körperkonzeptskala, FKKS), the Short Form 36 Health Survey (SF-36), the Beck Depression Inventory (BDI), the Social Phobia Inventory (SPIN) and the Social Interaction Anxiety Scale (SIAS). Patients were also scored with the Hamilton Depression Rating Scale (HDRS). SF-36 and FKKS data were compared to an age- and sex-matched healthy control group (*n* = 25) (13 men aged 53.7 ± 7.3 and 12 women aged 51.4 ± 5.2, mean ± SEM).

All patients gave written informed consent to participate. The study was approved by the ethics committee of Hannover Medical School (No. 6307).

### 2.1. Frankfurt Body Concept Scale (FKKS)

The FKKS [20,21,22,23] is a self-rating instrument that assesses different aspects of body concept of a person according to the agreement with several given statements. It represents one aspect of body image and might be defined as a developed conception of body that includes information from a subject’s perceptual experience (body percept) of his/her own body and the subject’s cognitive understanding (mythical or scientific knowledge) of the body in general [24]. It consists of nine subscales with a total of 64 items: (1) general health (6 items), (2) body care and taking care of body functioning (8 items), (3) physical efficacy (10 items), (4) body contact (6 items), (5) sexuality (6 items), (6) self-acceptance of one’s body (6 items), (7) acceptance of one’s body by others (4 items), (8) physical appearance (14 items) and (9) dissimilating body processes (4 items). The level of agreement with each statement is rated on a six-point scale [20]. Higher scores indicate a better body concept/body image.

### 2.2. Short Form 36 Health Survey (SF-36)

The SF-36 was developed to estimate the health-related quality of life in medical research [25,26]. It is a multi-item scale that evaluates both physical and mental components of functioning on a scale of 0 to 100. Higher scores are related to better quality of life. It is comprised of eight subscales: (1) physical functioning, (2) physical role functioning, (3) bodily pain, (4) general health perceptions, (5) vitality (energy and fatigue), (6) social functioning, (7) emotional role functioning and (8) mental health [25,27].

### 2.3. Beck Depression Inventory (BDI)

The BDI is a self-rating depression inventory consisting of 21 items that cover affective and somatic symptoms of depression [28]. Scores between 0 and 9 represent no depression, 10–18 mild depression, 19–29 moderate to severe depression and scores from 30 to 63 indicate severe depression. The test is designed in a way that patients have to define how they felt in the past week.

### 2.4. Hamilton Depression Rating Scale (HDRS)

The HDRS is a semi-structured interview that is performed by a healthcare professional. It consists of 21 items evaluating somatic symptoms and relatively few affective and cognitive symptoms of depression [29]. Scores of 0–7 indicate no depression, 8–16 mild depression, 17–23 moderate depression and 24 or higher severe depression [30].

### 2.5. Social Phobia Inventory (SPIN)

The SPIN is a 17-item self-rating scale for social anxiety disorder (social phobia). It includes 17 items assessing symptoms of social anxiety disorder (fear, avoidance, physiological arousal) [31]. Scores less than 20 represent no social phobia, 21–30 mild, 31–40 moderate, 41–50 severe and 51 and above very severe social phobia.

### 2.6. Social Interaction Anxiety Scale (SIAS)

The SIAS estimates anxiety during the interaction with others (friends, opposite sex, strangers). This includes fears of being inarticulate, sounding boring or stupid, not knowing how to respond and being ignored [32]. It includes 20 items. Scores higher than 43 indicate probable social interaction anxiety.

### 2.7. Statistical Analysis

**Figure 1:**
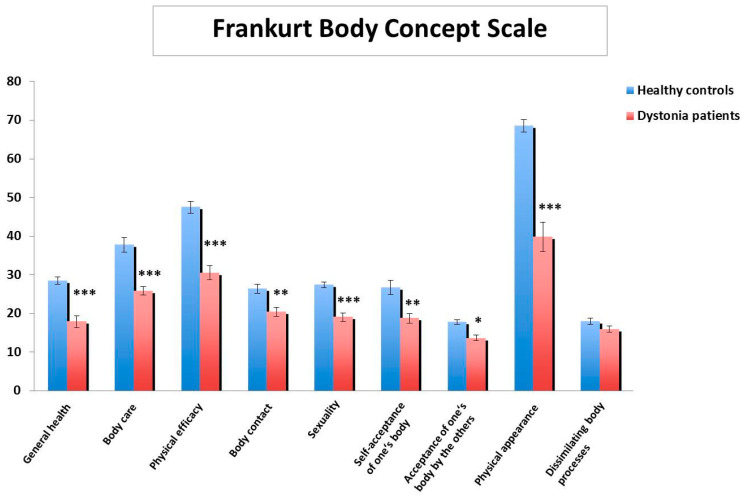
Frankfurt Body Concept Scale subscale scores in patients with dystonia (*n* = 20) in comparison to healthy controls (*n* = 25) indicate significant impairment in patients with dystonia in most domains; scores are presented as means ± SEM. * *p* < 0.05, ** *p* < 0.01, *** *p* < 0.001.

Descriptive analysis was conducted for all parameters. The Shapiro–Wilk test was used to test the normality. Non-normally distributed parameters were body care and taking care of body functioning (FKKS), sexuality (FKKS), physical role functioning (SF-36) and mental health (SF-36). The Mann–Whitney U Test was used to compare the SF-36 and FKKS subscale scores between patients with dystonia and the healthy control group. Correlations between dystonia motor scores (BFM and TWSTRS) and other scores (BDI, HDRS, SIAS, SPIN, SF-36 and FKKS) as well as between depression and anxiety scales and all subscores for SF-36 and FKKS were analyzed with the Pearson correlation test (Table 1 and Table 2). Pearson correlations (r) were also conducted to examine the associations between SF-36 and FKKS subscale scores (Figure 1). Multiple regression analysis was performed to elucidate which modalities of SF-36, BDI and HDRS have a higher impact on body concept. The factors affecting body concept were identified by using multiple regression models with the FKKS subscales as dependent variables and the SF-36, BDI and HDRS as independent variables. For statistical analyses and visualisation of the data we used SPSS (IBM Deutschland, Ehningen, Germany) and Excel (Microsoft Corp., Redmond, WA, USA). Data are presented as mean ± SEM; *p*-values < 0.05 were considered statistically significant. As this is an exploratory study, we did not correct the significance level for multiple comparisons.

## 3. Results

The mean BFM motor score of all 20 patients with dystonia was 19.4 ± 2.5, and the mean TWSTRS score of the seven patients with cervical dystonia was 8.6 ± 1.1. The mean MMST was 28.2 ± 1.2. Scores of the BDI and the HDRS indicated mild depression (12.0 ± 3.2 and 8.1 ± 2.1, respectively), and results of the SPIN showed mild social phobia (21.5 ± 3.6); however, social interaction anxiety scores were not increased (21.3 ± 4.9 on the SIAS).

Body concept as perceived by the patients was significantly impaired in eight domains compared to the healthy controls (Figure 1). The most pronounced differences between patients and controls were found for the subscales general health, body care and taking care of body functioning, physical efficacy, sexuality and physical appearance (all *p* < 0.001).

**Figure 2:**
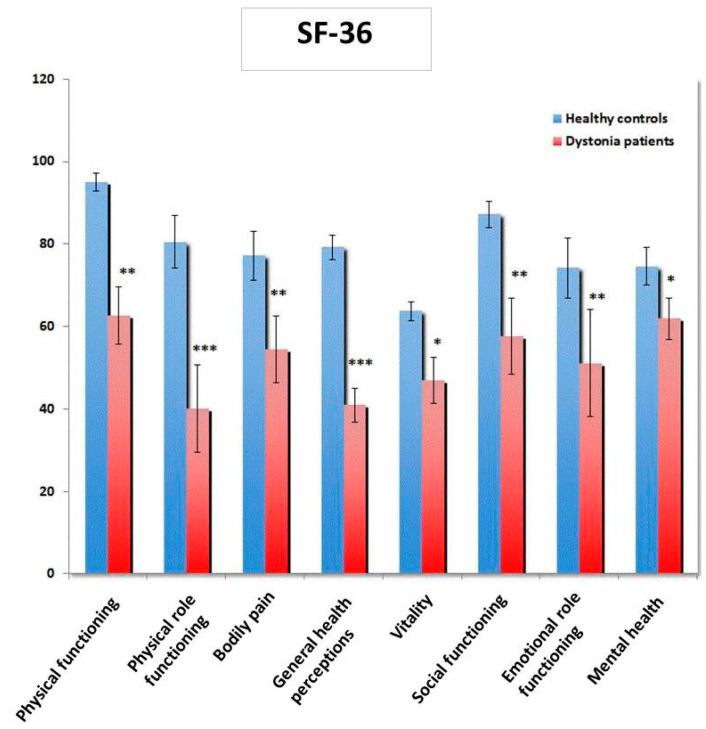
Quality of life (Short Form 36—SF-36) is reduced in patients with dystonia (*n* = 20) in comparison to healthy controls (*n* = 25); scores are presented as means ± SEM. * *p* < 0.05, ** *p* < 0.01, *** *p* < 0.001.

Similarly, patients with dystonia reported lower scores on all SF-36 subscales compared to the control group (Figure 2). The lowest scores were found for physical role functioning (*p* < 0.001) and general health perception (*p* < 0.001).

Table 1 summarizes Pearson’s correlations between BFM and TWSTRS and all other scales. The severity of motor symptoms (BFM but not TWSTRS) showed a significant correlation with both depression scale scores (HDRS, *p* = 0.008, r = 0.7; BDI, *p* = 0.04, r = 0.34). There were no significant associations between the disease severity scores and any of the SF-36 and Frankfurt Body Concept Scale subscale scores or the social anxiety scale scores.

In the next step we determined how the levels of depression and social anxiety were associated with quality of life and body concept. For this purpose, depression and anxiety scales were correlated with the subscales of the SF-36 and the FKKS. The BDI showed significant negative correlations with subscales of the SF-36 (physical role *p* = 0.004, r = −0.69; bodily pain *p* = 0.03, r = −0.54; social functioning *p* = 0.006, r = −0.67; emotional role *p* = 0.017, r = −0.61 and mental health *p* = 0.001, r = −0.76) and of the FKKS (sexuality *p* = 0.04, r = −0.44 and physical appearance *p* = 0.021, r = −0.73). Using the HDRS we also found significant negative correlations with subscales of the SF-36 (physical role *p* = 0.046, r = −0.54; bodily pain *p* = 0.026, r = −0.59 and mental health *p* = 0.05, r = −0.53) but not with the FKKS. None of the social anxiety scales was significantly correlated to subscales of the SF-36 or FKKS (Table 2).

**Figure 3:**
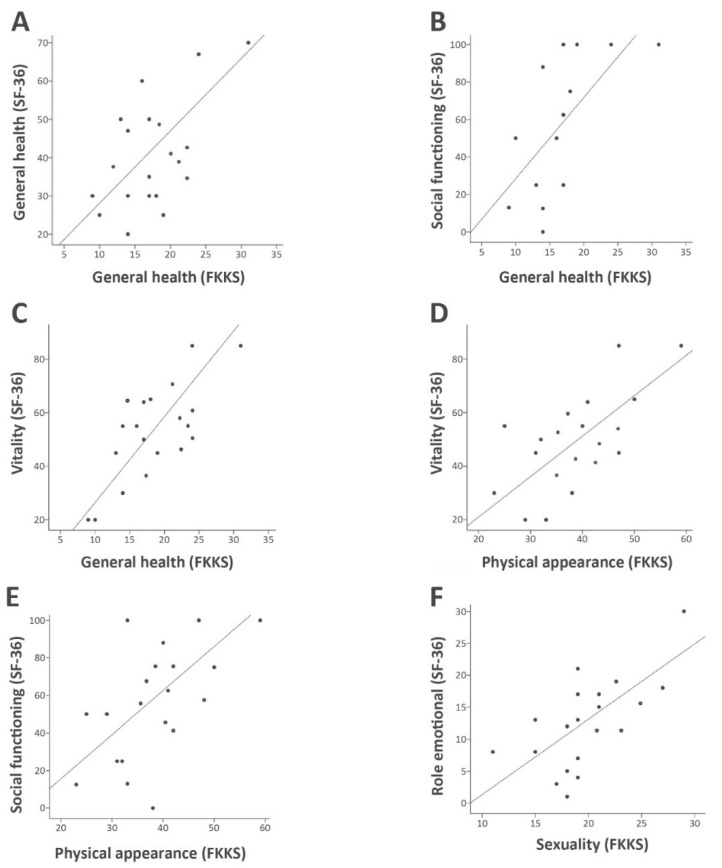
Correlations of SF-36 and FKKS subscales (Pearson correlation, *p* < 0.05, *n* = 20). (**A**) Correlation of self-perception of general health with general health (r^2^ = 0.41); (**B**) Correlation of self-perception of general health with social functioning (r^2^ = 0.42); (**C**) Correlation of self perception of general health with vitality (r^2^ = 0.65); (**D**) Correlation of physical appearance with vitality (r^2^ = 0.49); (**E**) Correlation of physical appearance with social functioning (r^2^ = 0.42); (**F**) Correlation of sexuality with emotional role functioning (r^2^ = 0.44).

In order to determine if body concept aspects are related to quality of life, the FKKS subscales were correlated with the SF-36 subscales. This revealed that the FKKS subscale general health was significantly associated with the SF-36 subscales general health (*p* = 0.01, r = 0.64), social functioning (*p* = 0.01, r = 0.65) and vitality (*p* = 0.04, r = 0.836). The FKKS subscale physical appearance (SASE) was positively correlated with the SF-36 subscales vitality (*p* = 0.005, r = 0.7) and social functioning (*p* = 0.01, r = 0.65). Low self-perceived sexuality in the FKKS was correlated with limited emotional functioning in SF-36 (*p* = 0.03, r = 0.53, Figure 3). All other correlations between the subscales were not significant.

The multiple regression analysis was determined to investigate which impairment of quality of life, BDI and HDRS mainly contributed to lower body concept. The vitality subscore in SF-36 had the strongest contribution to the general health impairment in FKKS (standardized beta = 0.74, *p* < 0.001), followed by the SF-36 subscore social functioning (standardized beta = 0.36, *p* < 0.001). Physical appearance in FKKS was uniquely contributed by vitality in SF-36 (standardized beta = 0.7, *p* = 0.005), whereas sexuality in FKKS was associated with the BDI (standardized beta = 0.71, *p* = 0.007) and the subscore emotional role in SF-36 (standardized beta = 0.52, *p* = 0.021). In the multiple regressions analysis HDRS lost its contribution to the body concept.

## 4. Discussion

In addition to previous studies that showed patients with dystonia may have depression, anxiety and reduced quality of life [5,7], our study indicates that aspects of body concept correlate both with depression and quality of life. Such correlations may be even more relevant than the mere severity of dystonia as assessed by standard rating scale.

In accordance with previous studies [10,33], we found a significantly reduced quality of life in patients with dystonia compared to healthy controls concerning both physical as well as mental aspects. In several studies a higher severity of dystonia is correlated with a lower quality of life. Page et al. [4] demonstrated that the patients with generalized dystonia have a lower quality of life than patients with focal dystonia. Although in previous studies depression and anxiety paralleled the impairment in quality of life [4,12,34], we found negative correlations of quality of life with depression scales only and not with anxiety scores. This may be due to the fact that we did not assess generalized anxiety aspects but specifically focused on social anxiety.

Body concept is a multidimensional construct that relates to a person’s thoughts, feelings and beliefs about his/her own body. It incorporates not only the estimation of body size but also the evaluation of body attractiveness and related thoughts and feelings [35,36]. We found a severely impaired self-evaluation of different components of the body concept in patients with dystonia compared to healthy controls. The attitude toward patients’ bodily wellbeing was decreased. In addition, our patients perceived themselves as disabled in taking care of their body and in improving its functionality, indicating a lack of attention to and consideration of the body. The strong attitude about the lack of health and bodily efficiency was one of the strongest “negative cognitions” of body concept. As thus it reflects awareness of physical appearance. Concepts about the esthetical appearance of the body as well as the perception of acceptance of the body by oneself and by others was significantly impaired in patients with dystonia. Furthermore, physical contact with others was judged uncomfortable. The affective dimension of the attitude toward one’s own attractiveness and sexuality was low in patients with dystonia. The aesthetic aspects of their appearance made their body unacceptable to them, which expresses impairments of more emotionally based aspects of body concept.

Interestingly, there was a significant correlation between self-rated depression and the FKKS subscales sexuality and physical appearance, which suggests that the self-perceived dissatisfaction with body image in patients with dystonia may partially be related to depression. Jahanshahi and Mardsen [13] found that a more negative body concept as well as self-blame, self-accusation and self-punishment were prominent components in patients with cervical dystonia. This previous study applied the Body Concept Scale (BCS) consisting of 22 differential seven-interval semantic scales and an 11-point rating scale indicating the degree of self-perceived physical disfigurement [13]. Of note, in our study using the more detailed FKKS, the impaired body concept was not associated with an anxiety disorder or the severity of dystonia. This indicates that the impaired body concept is neither disease-severity dependent nor is it prominently associated with social anxiety.

Remarkably, several components of body concept interfere with quality of life in our patients. Decreased perception of bodily wellbeing, sexuality and aspects of physical appearance were positively correlated with the physical and psychological subscale scores of the SF-36, such as general health, social functioning, vitality and emotional wellbeing, suggesting that quality of life in dystonia patients is inflicted by perceived internal body concept conflicts.

Several limitations of our study have to be considered. First of all, the study had a cross-sectional design, which did not allow drawing causal conclusions. The study population included a limited number of patients. Even though we matched the control group with regard to age and sex, we did not assess weight and body mass index (BMI), which might have influenced the body concept and should be mentioned in future studies. Moreover, the controls were a group of healthy subjects but not patients with chronic condition. Furthermore, patients who are determined to undergo surgery might have different impairments than those who wish to continue conservative therapy.

Overall, our study demonstrates an additional aspect of the psychosocial burden of dystonia. According to our findings, dystonia is accompanied by an impaired body concept that is associated with impaired quality of life and mild levels of depression. The score of the body concept scale FKKS after deep brain stimulation (DBS) will be an interesting addition for the understanding of the findings. The impact of therapeutics on the body concept impairment in patients with dystonia remains to be elucidated.

## Figures and Tables

**Table 1:** Correlations of motor scores in BFM (Burke–Fahn–Marsden Dystonia Rating Scale) and TWSRTS (Toronto Western Spasmodic Torticollis Rating Scale) with all other scales (Pearson correlation test, * *p* < 0.05, ** *p* < 0.01).

	BFM (*n* = 20)		TWSTRS (*n* = 7)	
	Pearson r	*p*-Value	Pearson r	*p*-Value
**Mood scales**				
Beck Depression Inventory	0.34	0.04 *	0.43	*0.21*
Hamilton Depression Rating Scale	0.70	0.008 **	0.52	0.08
**Anxiety scales**				
Social Phobia Inventory	0.21	0.30	0.76	0.12
Social Interaction Anxiety Scale	0.67	0.43	0.16	0.29
**Frankfurt Body Concept Scale**				
General health	0.08	0.79	−0.62	0.54
Body care and taking care of body functioning	0.27	0.35	0.48	0.11
Physical efficacy	0.34	0.22	0.23	0.09
Body contact	−0.14	0.96	0.56	0.16
Sexuality	0.16	0.57	0.26	0.09
Self-acceptance of one’s body	−0.15	0.62	0.14	0.15
Acceptance of one’s body by others	0.35	0.15	−0.28	0.52
Physical appearance	−0.23	0.32	0.35	0.42
Dissimilating body processes	0.21	0.44	0.21	0.08
**SF-36 Quality of life scale**				
Physical functioning	0.42	0.23	−0.22	0.17
Role physical	0.34	0.57	0.42	0.54
Bodily pain	0.23	0.66	−0.35	0.54
General health	−0.14	0.09	0.18	0.36
Vitality	0.23	0.65	0.24	0.09
Social functioning	0.34	0.72	0.52	0.12
Role emotional	0.43	0.08	0.22	0.11
Mental health	0.39	0.66	0.32	0.31

**Table 2:** Correlations of depression (Beck Depression Inventory—BDI, Hamilton Depression Rating Scale—HDRS) and social anxiety scales (Social Phobia Inventory—SPIN, Social Phobia Interaction Anxiety Scale—SIAS) with quality of life (SF-36) and the Frankfurt Body Concept Scale (FKKS), * *p* < 0.05, ** *p* < 0.01 (*n* = 20).

	BDI		HDRS		SPIN		SIAS	
	Pearson r	*p*-Value	Pearson r	*p*-Value	Pearson r	*p*-Value	Pearson r	*p*-Value
**Quality of life scales**								
Physical functioning	−0.49	0.09	−0.31	0.29	−0.46	0.10	−0.02	0.94
Role physical	−0.69	0.004 **	−0.54	0.046 *	−0.13	0.66	0.27	0.32
Bodily pain	−0.54	0.03 *	−0.59	0.026 *	−0.65	0.11	−0.14	0.61
General Health	−0.23	0.41	0.08	0.79	0.15	0.39	−0.19	0.50
Vitality	−0.42	0.12	−0.02	0.94	−0.38	0.17	0.03	0.91
Social functioning	−0.67	0.006 **	−0.43	0.12	−0.08	0.80	0.52	0.15
Role emotional	−0.60	0.017 *	−0.28	0.34	−0.18	0.53	0.11	0.69
Mental Health	−0.76	0.001 **	−0.53	0.05 *	−0.40	0.15	−0.10	0.72
**Frankfurt Body Concept Scale**								
General health	0.36	0.17	−0.14	0.61	0.27	0.31	0.06	0.82
Body care and taking care of body functioning	−0.10	0.71	−0.37	0.18	0.12	0.67	0.18	0.50
Physical efficacy	0.11	0.67	−0.40	0.13	0.33	0.21	0.61	0.11
Body contact	0.20	0.46	−0.11	0.69	0.42	0.10	−0.10	0.73
Sexuality	−0.44	0.04 *	0.01	0.98	0.31	0.25	0.08	0.76
Self−acceptance of one’s body	0.09	0.72	−0.32	0.24	0.04	0.88	0.55	0.23
Acceptance of one’s body by others	0.03	0.91	−0.07	0.80	0.03	0.92	0.05	0.86
Physical appearance	−0.73	0.021 *	−0.50	0.06	−0.15	0.57	0.29	0.27
Dissimilating body processes	−0.07	0.79	−0.02	0.92	0.08	0.77	0.02	0.94
